# Cortical Sources of Respiratory Mechanosensation, Laterality, and Emotion: An MEG Study

**DOI:** 10.3390/brainsci12020249

**Published:** 2022-02-11

**Authors:** Pei-Ying S. Chan, Chia-Hsiung Cheng, Chia-Yih Liu, Paul W. Davenport

**Affiliations:** 1 Department of Occupational Therapy and Healthy Aging Research Center, Chang Gung University, Taoyuan 333, Taiwan; 2 Department of Psychiatry, Chang Gung Memorial Hospital, Taoyuan 333, Taiwan; liucy752@cgmh.org.tw; 3 BIND Lab, Chang Gung University, Taoyuan 333, Taiwan; 4 Department of Physiological Sciences, College of Veterinary Medicine, University of Florida, Gainesville, FL 32611, USA; pdavenpo@ufl.edu

**Keywords:** respiratory sensation, respiratory-related evoked field (RREF), respiratory-related evoked potential (RREP), magnetoencephalography (MEG), dyspnea

## Abstract

Airway obstruction activates mechanoreceptors that project to the cerebral cortices in humans, as evidenced by scalp encephalography recordings of cortical neuronal activation, i.e., respiratory-related evoked potential (RREP). However, neural evidence of both high spatial and temporal resolution of occlusion-elicited cortical activation in healthy individuals is lacking. In the present study, we tested our hypothesis that inspiratory mechanical stimuli elicit neural activation in cortical structures that can be recorded using magnetoencephalography (MEG). We further examined the relationship between depression and respiratory symptoms and hemispheric dominance in terms of emotional states. A total of 14 healthy nonsmoking participants completed a respiratory symptom questionnaire and a depression symptom questionnaire, followed by MEG and RREP recordings of inspiratory occlusion. Transient inspiratory occlusion of 300 ms was provided randomly every 2 to 4 breaths, and approximately 80 occlusions were collected in every study participant. Participants were required to press a button for detection when they sensed occlusion. Respiratory-related evoked fields (RREFs) and RREP peaks were identified in terms of latencies and amplitudes in the right and left hemispheres. The Wilcoxon signed-rank test was further used to examine differences in peak amplitudes between the right and left hemispheres. Our results showed that inspiratory occlusion elicited RREF M1 peaks between 80 and 100 ms after triggering. Corresponding neuromagnetic responses peaked in the sensorimotor cortex, insular cortex, lateral frontal cortex, and middle frontal cortex. Overall, the RREF M1 peak amplitude in the right insula was significantly higher than that in the left insula (*p* = 0.038). The RREP data also showed a trend of higher N1 peak amplitudes in the right hemisphere compared to the left (*p* = 0.064, one-tailed). Subgroup analysis revealed that the laterality index of sensorimotor cortex activation was significantly different between higher- and lower-depressed individuals (−0.33 vs. −0.02, respectively; *p* = 0.028). For subjective ratings, a significant relationship was found between an individual’s depression level and their respiratory symptoms (Spearman’s rho = 0.54, *p* = 0.028, one-tailed). In summary, our results demonstrated that the inspiratory occlusion paradigm is feasible to elicit an RREF M1 peak with MEG. Our imaging results showed that cortical neurons were activated in the sensorimotor, frontal, middle temporal, and insular cortices for the M1 peak. Respiratory occlusion elicited higher cortical neuronal activation in the right insula compared to the left, with a higher tendency for right laterality in the sensorimotor cortex for higher-depressed rather than lower-depressed individuals. Higher levels of depression were associated with higher levels of respiratory symptoms. Future research with a larger sample size is recommended to investigate the role of emotion and laterality in cerebral neural processing of respiratory sensation.

## 1. Introduction

Breathing is an interoceptive process, and eupneic breathing works effortlessly. Sensory receptors, including mechanoreceptors, chemoreceptors, and lung vagal afferents, receive respiratory neural input from the lungs and airways [1,2]. This periphery unceasingly provides sensory information to the brain, which takes up its role in discriminative processing and affective processing of different types of stimuli, e.g., mechanical loads, bronchoconstriction, hyperinflation, and blood gas changes [3,4]. Although researchers do not fully understand all cortical and subcortical neural pathways involved in respiratory sensory processing, previous studies have provided evidence related to respiratory-related brain activations in both humans and animals [5,6,7,8,9,10,11,12,13,14,15,16,17,18]. For example, a respiratory-related evoked potential (RREP) was recorded in lamb cortex [10], and phrenic nerve stimulation elicited an evoked potential in cat cerebral cortex [15]. In human electroencephalogram (EEG) measurements, researchers have studied respiratory occlusion-elicited potential, i.e., respiratory-related evoked potential (RREP), to understand respiratory mechanosensory processing in the higher cortex [18,19]. RREP consists of early components Nf, P1, and N1 (<160 ms post-trigger) and late components P2 and P3 (>180 ms post-trigger) [2,17].

The RREP method has excellent temporal resolution but lower spatial resolution when studying the neural substrates involved in cerebral processing of respiratory sensation. Therefore, functional magnetic resonance imaging (fMRI) is often used to compensate for the shortcomings of EEG measurements. Past fMRI studies have revealed that short inspiratory occlusion elicits brain neural activation in brain substrates, including cortical (sensorimotor cortices, frontal lobes, supramarginal gyrus, cingulate cortex) and subcortical (thalamus, hippocampus, and caudate) areas [20,21,22]. Other neuroimaging studies have also shown that dyspnea induced by resistive load is associated with neural activation in the amygdala, cingulate cortex, and insular cortex [7,9,23,24].

Results regarding hemispheric differences in respiratory sensation have been mixed in RREP studies. Some researchers have discovered that RREP peak amplitudes tend to be greater in the right cortex compared to the left [19], while others have not found much difference between the two sides [10,25]. Some past attempts examining the effect of negative emotion (e.g., anxiety) on RREP have not found significant differences in terms of peak amplitudes between the two hemispheric recordings [26,27]; however, with the fMRI technique, a recent study did notice an association between anxiety level and activation of the precuneus and inferior parietal gyrus in the right hemisphere in response to short inspiratory occlusion [21]. Additionally, results in the literature regarding emotional influences on hemispheric lateralization are mixed [28,29,30]. Generally, two types of theoretical models have been proposed in the literature. The first is related to hemispheric specialization of different emotions, where the right hemisphere is thought to process negative emotions and the left hemisphere for positive emotions [31,32]. For example, in a study focusing on emotional picture stimulus processing, researchers found that participants tended to respond to the left side for negative emotional expression pictures and to the right side for positive emotional expression pictures [32]. The second model suggests dominance of the right hemisphere in processing all emotions [33,34,35,36]. For instance, the magnitude of the startle reflex (associated with fear emotions) was suggested to be processed mainly by the right hemisphere, while weak processes were found in the left hemisphere [30]. Similarly, in a review study conducted by Hecht (2010), it was suggested that depression is highly associated with a hyperactive right hemisphere and that the right hemisphere is more sensitive to stress and pain adversity compared to the left hemisphere [37].

fMRI measurements are limited in temporal resolution and unable to reflect corresponding changes in milliseconds as in RREP. The magnetoencephalography (MEG) technique is known to have both temporal and spatial resolution in brain activation and information processing and may be an appropriate choice for studying central processing of respiratory sensation. Several studies have suggested that MEG is one of the most efficient tools for investigating source localization with a priori hypotheses [38,39]. Therefore, the primary aim of the present study was to test the feasibility of using MEG to examine brain activation in response to short inspiratory occlusion. Based on previous findings in fMRI and RREP studies, we hypothesized that transient occlusion elicits cortical activation in the bilateral frontal cortex, sensorimotor cortex, temporal cortex, and insular cortex. We further hypothesized that the RREP peak and the respiratory-related evoked fields (RREFs) generated by MEG recordings in the right hemisphere show greater amplitudes compared to the left hemisphere in response to respiratory occlusion. In addition, in the present study, we tested the relationship between depression status and self-reported respiratory symptoms, as well as hemispheric dominance. We hypothesized that depression is associated with increased level of self-reported breathlessness and right-hemispheric dominance in response to short inspiratory occlusion.

## 2. Methods

### 2.1. Study Participants

Healthy nonsmoking adults of at least 20 years of age without self-reported respiratory, cardiovascular, and/or neurological diseases were eligible to participate in this study. Additionally, potential subjects needed to comply with MEG scanner safety requirements in order to be recruited. All subjects were instructed to refrain from a large meal, caffeine, and exercise for at least an hour before starting the neuromagnetic or RREP experiment. The study procedures were approved by the Institutional Review Board of the Chang Gung Medical Foundation (IRB Approval Code: 104-6002B).

### 2.2. Respiratory Apparatus

Each subject was required to breathe through a face mask (Hans Rudolph, Inc.) connected to a two-way non-rebreathing valve (Hans Rudolph, Inc.). The inspiratory port of the breathing valve was connected to a pneumotachograph amplifier (1110 series, Hans Rudolph, Kansas City, MI, USA) and a customized occlusion valve (Hans Rudolph) external to the scanner. The occlusion valve was connected to a pressure tank and an external trigger via a solenoid. Mouth pressure, tidal volume, breathing frequency, minute ventilation, and inspiratory time were recorded with differential pressure transducers (Hans Rudolph) and continuously monitored during the experiment (LabChart 7, Powerlab Inc., Bella Vista, Australia).

### 2.3. Experiment Procedure

All participants were provided with a detailed informed consent form with explanations regarding the nature of the study. After consenting, participants completed the self-rated respiratory symptom questionnaire designed by our laboratory, based on a modification of St. George’s Respiratory Questionnaire [40]. They were also instructed to complete the Chinese version of the Beck Depression Inventory-II (BDI-II) [41]. Our respiratory symptom questionnaire has 6 sections of questions examining subjects’ experience of respiratory symptoms during daily living, and the total scores range from 0 to 113 (higher scores indicating higher levels of respiratory symptoms). Participants were then instructed to perform a pulmonary function test. Participants were required to have their forced expiratory volume in 1 s (FEV1) at least 70% of the predicted values in order to proceed with the experiment. After the screening, participants were instructed to sit in the scanner with their heads immobilized and wear the face mask in position along with the connected respiratory apparatus. Participants were familiarized with the breathing apparatus for approximately 30 s before recordings. During the experiment, each participant was instructed to manually signal detection when they sensed the occlusions.

For neuromagnetic recordings, data acquisition was performed using a 306-channel MEG system (Elekta Neuromag^®^ TRIUX) with a sampling rate of 1 KHz and an online bandpass filtered at 0.3–30 Hz. Data from 204 planar gradiometers were used for analysis, because such gradiometers detect the largest signal right above the activated brain areas. Four head indicator coils were placed on the scalp, and their locations were detected by measuring the magnetic fields produced by the currents fed into them. The coil locations in relation to anatomic landmarks (i.e., left preauricular point, right preauricular point, and nasion) were determined with a 3D digitizer. The online recording bandpass was 0.1–1 KHz at a sampling rate of 1 KHz. Electro-oculograms (EOGs) monitoring eye movements were recorded. Epochs contaminated by eye blinks (EOG > 100 μV) were removed. The respiratory apparatus located inside the shielded room was all nonmagnetic. Inspiratory occlusions were performed by the experimenter by manually closing the occlusion valve via an external trigger located external to the scanner. The RREP experiment protocol was the same as described above for the neuromagnetic experiment, and the two experiments were scheduled at least 2 days apart. For the RREP experiment setups, refer to the previous study described by Chan et al. (2014) [26].

### 2.4. Data Analysis

Data reduction and analysis were performed using Brainstorm software (version 3.4) for the neuroimaging data. Before preprocessing, gradiometer channels with excessive amplitudes (higher than 3000 fT) were identified and excluded from the analysis. After using signal space projections to correct artifacts from eye blinks, a bandpass filter with 0.1–30 Hz on the evoked magnetic field, i.e., respiratory-related evoked field (RREF), was applied. MEG recordings of single-trial RREF consisted of a 100 ms baseline (pre-triggering) with a duration of 1 s after trigger onset. At least 70 artifact-free trials were averaged.

The forward model was computed using the overlapping sphere method based on the MNI/Colin27 template in Brainstorm software. Cortical sources of the event-related magnetic fields were estimated using wMNE source modeling with constraints on source orientation. The MNE method is an efficient tool for functional mapping, and the number of dipoles used for source estimation was approximately 15,000. For group analysis, we utilized the Desikan and Killiany Atlas as a template to scout regions of interest (ROIs). Based on the findings in the literature, in the present study, we selected the frontal lobe, temporal gyrus, sensorimotor cortex, and insular cortex as ROIs [4,42]. The magnitude of each dipole was normalized to its baseline level, and the *z*-scores were extracted for every ROI. Laterality index (LI) was also calculated for ROIs. For RREP peaks, P1 peak amplitudes were extracted from the CP3 and CP4 electrodes and N1 peak amplitudes from the C3 and C4 electrodes. The Wilcoxon signed-rank test was performed for RREP peaks (one-tailed based on the a priori hypothesis) and also for four RREF ROIs to compare RREF peak amplitudes between the right and left hemispheres (SPSS 19.0). Since the M1 peak amplitudes between the left and right hemispheres were compared in the four ROIs, the significance level was therefore adjusted for multiple comparisons using the Benjamini and Hochberg approach [43], and the false discovery rate (FDR) was set to 0.15. Along with the original *p*-value, another FDR *p*-value was presented. Additionally, Spearman’s rank correlation analysis was performed to examine the relationship between the BDI-II scores and participants’ respiratory symptom questionnaire scores. According to the scoring criteria of the BDI-II, the score ranges from 0 to 63, where scores of over 13 are indicative of mild depressive status [41]. Due to the fact that the 14 participants in the current study were not diagnosed with any clinical depression and only presented with a minimal level of depression (if any), our subgroup analysis only applied an average split on the sample for grouping purposes. A subsequent subgroup analysis was performed to examine the difference in hemispheric dominance in terms of LI between higher- and lower-depressed individuals. The threshold for statistical significance was set at *p* < 0.05.

## 3. Results

Participants’ demographic, PFT data, and subjective ratings are presented in Table 1. All participants passed the minimum requirement of the pulmonary function test in order to continue the experiment.

**Figure 1:**
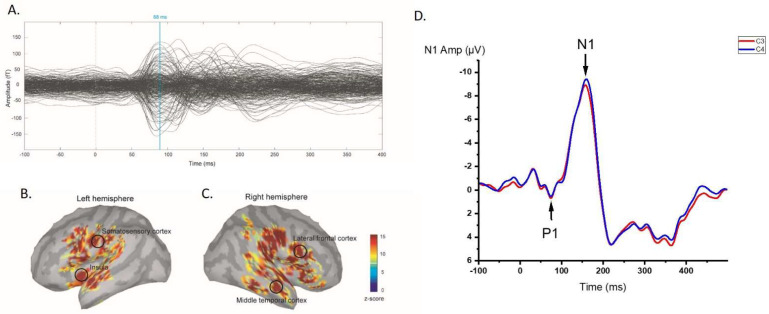
(**A**) Group-averaged RREF waveforms for 14 individuals; an M1 peak was identified approximately 80–100 ms after trigger onset; (**B**,**C**) averaged neural activation map with 4 ROIs presented for 14 individuals; (**D**) group-averaged RREP P1 and N1 peak waveforms in left and right hemispheres, represented by C3 (red line) and C4 (blue line) electrodes, respectively.

An RREF M1 peak was identified at approximately 80–100 ms after onset of trigger. Figure 1 shows the activation map of the effect of inspiratory occlusions for the group of 14 individuals.

**Figure 2:**
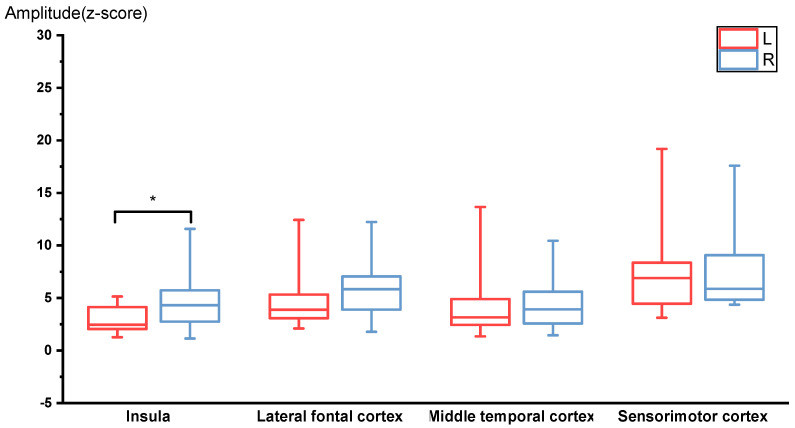
RREF M1 peak amplitudes for the 4 ROIs, including insula, lateral frontal cortex, middle temporal cortex, and sensorimotor cortex (N = 14). The right insula was activated significantly higher compared to the left insula. * *p* < 0.05.

Significant activation was observed in the sensorimotor cortices, middle temporal cortices, and insular cortices at the RREF M1 peak. The mean amplitudes of the M1 peak in the sensorimotor cortices, middle temporal cortices, insular cortices, and lateral frontal cortices were 7.40 ± 4.25 fT and 8.07 ± 4.67 fT, 4.59 ± 3.82 fT and 4.36 ± 2.5 fT, 2.92 ± 1.21 fT and 4.69 ± 2.62 fT, 4.63 ± 2.58 fT, and 6.20 ± 3.32 fT for the left and right side, respectively. The Wilcoxon signed-rank test of the magnitude of activation between the left and right hemispheres revealed that the right insula was significantly more activated during the occlusion event (*p* = 0.028, two-tailed; significant after the Benjamini–Hochberg procedure with FDR = 0.088). Figure 2 shows a boxplot for the amplitudes measured for each ROI at the M1 peak. The laterality index for the RREF M1 peak in the sensorimotor cortex, lateral frontal cortex, insular cortex, and middle frontal cortex was −0.20 ± 0.30, −0.10 ± 0.34, −0.22 ± 0.32, and 0.17 ± 0.48, respectively.

**Figure 3:**
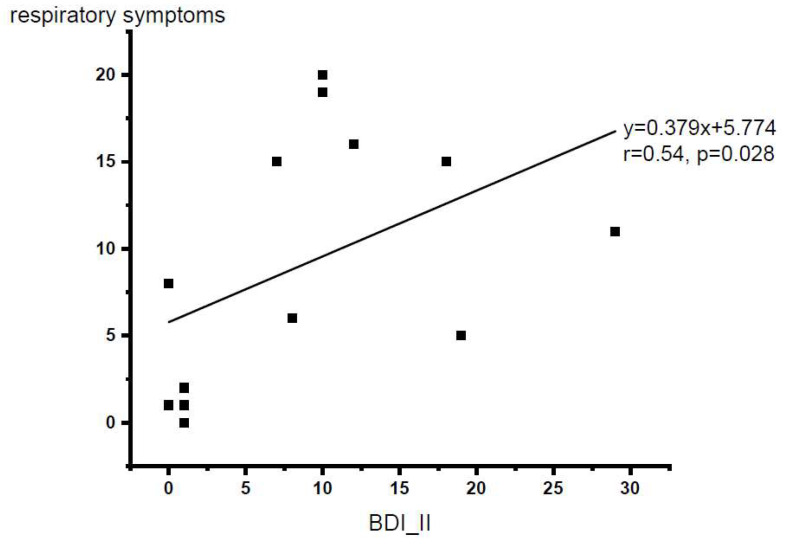
Scatter plot for the BDI-II scores vs. respiratory symptom questionnaire total scores (N = 13 with one outlier excluded). *p* < 0.05, one-tailed.

After removing one outlier (total respiratory symptom score of 35) from the database, Spearman’s rank correlation analysis demonstrated that there was a significant relationship between respiratory symptoms and depression level (Spearman’s rho = 0.54, *p* = 0.028, one-tailed). Figure 3 shows the scatter plot of depression level versus respiratory symptom level (N = 13).

**Figure 4:**
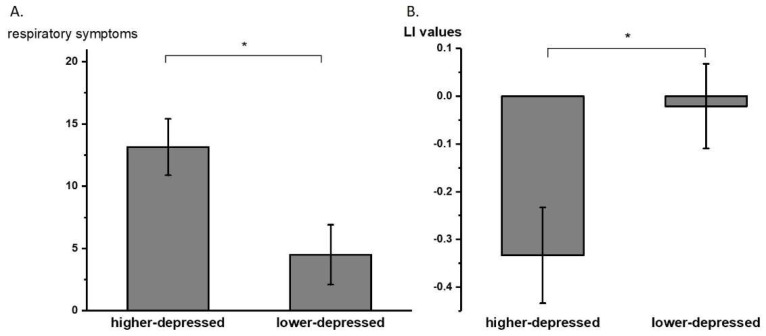
(**A**) Averaged (±SE) respiratory symptom questionnaire total scores of higher- and lower-depressed subgroups. (**B**) Averaged (±SE) laterality index (LI) values of higher- and lower-depressed groups with average split. * *p* < 0.05.

Subgroup analyses (with an average split of BDI-II scores M ± SD = 9.43 ± 8.76) using the Mann–Whitney U test revealed that the higher-depressed (BDI-II scores of 10 or higher, N = 7 after excluding one outlier value) group rated higher scores than the lower-depressed (BDI-II scores of less than 10, N = 6) group in our respiratory symptom questionnaire (*p* = 0.017). The laterality indices in the sensorimotor cortex between the higher-depressed (N = 8) group and the lower-depressed group (N = 6) were significantly different (−0.33 ± 0.28 and −0.02 ± 0.21, respectively; *p* = 0.028, significant after the Benjamini–Hochberg procedure with FDR = 0.112). Figure 4 shows the averaged LI values of the higher- and lower-depressed groups of individuals in the sensorimotor cortex.

The RREP peaks and amplitudes were also identified for the 14 subjects. The averaged P1 amplitude peaks in the centroparietal area were 3.25 ± 1.77 μV and 3.6 ± 1.89 μV for the CP3 and CP4 electrodes, respectively. The averaged N1 amplitudes peaked at the central area were −8.73 ± 3.69 μV and −9.51 ± 4.39 μV for the C3 and C4 electrodes, respectively. The P1 peak amplitude did not differ significantly between the left and right sides (*p* = 0.17, one-tailed); however, the N1 peak amplitudes showed a trend of being slightly larger in the right than that in the left side (*p* = 0.064, one-tailed).

## 4. Discussion

Our study demonstrated that transient inspiratory occlusions of 300 ms are feasible to elicit cortical neural responses, RREF, measured by MEG. A short-latency M1 peak was identified between 80 and 100 ms after trigger onset (approximately 50–70 ms after occlusion onset). The results showed initial cortical neural activation in the somatosensory cortices, middle temporal lobe, lateral frontal lobe, and insular cortices for the RREF M1 peak. Furthermore, our data demonstrated that neural activation in response to transient occlusions was significantly higher in the right insular cortex compared to the left. In addition, our data showed that an increased depression level was positively associated with increased respiratory symptoms. The subgroup analysis further revealed that higher-depressed individuals not only reported higher levels of dyspnea, but also had more tendency for right-hemispheric laterality in response to inspiratory occlusions compared to lower-depressed individuals.

The RREF M1 peak was identified at approximately 80–100 ms as the first consistent peak after onset of occlusion trigger. This M1 peak reflects the RREP P1 deflection temporally [17,26,44,45]. In terms of source localization, S1 cortex activation was consistent with previous RREP reports for P1 deflection [42,46]. With 100 ms inspiratory occlusion stimuli, Logie et al. (1998) discovered RREP source localizations for Nf and P1 peaks were radial dipoles, primarily oriented to the precentral and postcentral cortices by applying minimum norm estimate analysis [46]. Using a high-density 128-electrode EEG recording system, von Leupoldt et al. (2010) reported a negative band in the frontal area for the Nf peak and a positive band in the centroparietal area for the P1 peak [42]. Recent fMRI reports also suggested that bilateral sensorimotor cortices were consistently activated by transient 150 ms inspiratory occlusions [20,21].

In the present study, the M1 peak was identified because it was the first consistent peak analyzed for the 14 individuals, and no mid- and late-latency RREF peaks were analyzed in this dataset. Therefore, future MEG studies with a larger sample size are encouraged to analyze longer latency peaks in the RREF and localize neural sources for peaks corresponding to RREP N1, P2, and N2 peaks. The current dataset showed that the most prominent source at the M1 timepoint was at the sensorimotor cortex, while the insular cortex, lateral frontal cortex, and middle temporal cortex were activated with lower amplitudes. It is expected that in future replicated studies, when a later RREF peak corresponding to the RREP N1 peak is identified, higher levels of activation would be observed in the aforementioned areas for the second RREF peak.

The lateral frontal cortex in the middle frontal gyrus (MFG) was also found to be activated bilaterally. The present results are consistent with some past RREP and fMRI studies [4,20,21,42]. For example, von Leupoldt et al. (2010) suggested the right lateral frontal cortex as an area of origin of cortical sources for RREP [42]. In contrast, Davenport and Vovk (2010) reported that load elicited cortical activations primarily in the inferior frontal gyrus and opercula triangularis, and they identified MFG as a major area associated with chemoreception [4]. However, later fMRI studies consistently reported MFG as one major area of cortical neural activation by single and paired inspiratory occlusions [20,21]. Our experimental setup of transient inspiratory occlusion stimulus involved changing blood gas, which activated chemoreceptors for homeostasis. The role of MFG in occlusion-elicited cerebral respiratory sensation needs to be clarified further.

Our RREF data also demonstrated that the right insular cortex was activated significantly higher than the left insula. Although the insular cortex was not identified as a neural origin in past RREP source localization studies, it has been consistently considered a major brain substrate in dyspnea-related studies, especially for fMRI studies examining the relationship between aversive emotions and dyspneic sensations [6,8,9,23,24]. This supports Revelette and Davenport’s (1990) report of RREP, contrary to a few other past RREP studies [47].

In our earlier RREP studies examining anxiety effects on respiratory cerebral processing, a difference was hardly found between the left and right hemispheres [48,49,50]. Additionally, the inferior frontal gyrus has been identified as one area activated by inspiratory loading mostly bilaterally in past respiratory cortical neural activation studies [20,21,23]. However, another study suggested that there are distinguished effects between depression and anxiety on interoception of the heartbeat [51]. Parallel to our results, a past imaging study found participants’ right inferior parietal gyrus and right precuneus activation increased as their anxiety levels increased [21]. Other studies suggest that the right hemisphere is related to processing depression-related stimuli such as fear and stress [52,53]. Taken together, the above evidence supports that affective processing of respiratory information may be lateralized to the right hemisphere.

Finally, despite the small sample size, our results demonstrated that a higher depression level was associated with a higher level of dyspnea, as represented by our respiratory symptom questionnaire. With an average split of the depression questionnaire scores, our subgroup analyses further revealed that higher-depressed individuals reported more respiratory symptoms than lower-depressed individuals in their daily life. Past research has suggested that a negative affect is associated with higher bodily symptom reports [54]. Some even suggested that individuals with more negative emotions tend to be more accurate in the interoceptive discrimination task but show a more aversive response to ambiguous stimuli compared to those with less negative emotions [55]. Future studies with a larger sample size are recommended to examine the relationship between depression and respiratory interoceptive attention to dyspneic sensation. Furthermore, the calculated laterality index in the sensorimotor cortex showed that higher- compared to the lower-depressed individuals were approximately 15 times more lateralized to the right hemisphere. Again, this result is supported by past literature, where it has been suggested that emotion is highly associated with right-hemispheric dominance [35,37].

In conclusion, this study demonstrated that the inspiratory occlusion paradigm is feasible to elicit an RREF M1 peak with MEG recordings. Our imaging results showed that cortical neurons were activated in the sensorimotor, frontal, middle temporal, and insular cortices for the M1 peak. Our RREF data also showed that respiratory occlusions elicited higher cortical neuronal activations in the right insula compared to the left. In addition, higher-depressed individuals seem to process respiratory occlusions with a higher level of right laterality than lower-depressed individuals in the sensorimotor cortex. Future research is needed to investigate the role of emotion and laterality in cerebral neural processing of respiratory occlusion stimulus.

## Figures and Tables

**Table 1:** Baseline characteristics of the study participants (N = 14, mean ± SD).

Variables	All Subjects
*N*	14
Age (years)	23.66 ± 3.18
Gender (female/male)	7/7
FEV1 (liters)	3.19 ± 0.55
FEV1 (%) of predicted value	81.93 ± 10.13 (%)
Subjective respiratory symptom score	11.21 ± 10.15

## Data Availability

The data that supports the results of this study are available upon request and can be obtained from the corresponding authors.
